# Our Health Counts: Examining associations between colonialism and ever being incarcerated among First Nations, Inuit, and Métis people in London, Thunder Bay, and Toronto, Canada

**DOI:** 10.17269/s41997-023-00838-6

**Published:** 2023-12-29

**Authors:** Nicole M. Muir, Michael Rotondi, Raman Brar, Nooshin Khobzi Rotondi, Cheryllee Bourgeois, Brian Dokis, Michael Hardy, Raglan Maddox, Janet Smylie

**Affiliations:** 1 York University, Toronto, ON Canada; 2 Well Living House, Li Ka Shing Knowledge Institute, Unity Health Toronto, St. Michael’s Hospital, Toronto, ON Canada; 3 Faculty of Health Sciences, Ontario Tech University, Toronto, ON Canada; 4 Seventh Generation Midwives Toronto, Toronto, ON Canada; 5 Southwest Ontario Aboriginal Health Access Centre (SOAHAC), London, ON Canada; 6 Anishnawbe Mushkiki, Thunder Bay, ON Canada; 7 Research School of Population Health, Australian National University, Canberra, Australia

**Keywords:** Foster care, Incarceration, Victimization, Urban Indigenous health, Anti-Indigenous racism, Residential schools, Placement en famille d’accueil, incarcération, victimisation, santé des Autochtones en milieu urbain, racisme anti-Autochtones, pensionnats

## Abstract

**Objectives:**

Indigenous peoples have a disproportionately high prevalence of incarceration in the Canadian justice system. However, there is limited Indigenous-driven research examining colonialism and the justice system, specifically associations between racism, externally imposed family disruptions, and history of ever being incarcerated. Therefore, this study examined the association between the proportion of previous incarceration and family disruption, experiences of racism, and victimization for Indigenous adults in London, Thunder Bay, and Toronto, Ontario, Canada. The three communities expressed that they did not want comparison between the communities; rather, they wanted analysis of their community to understand where more supports were needed.

**Methods:**

Indigenous community partners used respondent-driven sampling (RDS) to collect data from First Nations, Inuit, and Métis (FNIM) peoples in London, Thunder Bay, and Toronto. Prevalence estimates, 95% confidence intervals, and relative risk were reported using unweighted Poisson models and RDS-adjusted proportions.

**Results:**

Proportions of ever being incarcerated ranged from 43.0% in London to 54.0% in Toronto and 72.0% in Thunder Bay. In all three cities, history of child protection involvement and experiencing racism was associated with an approximate 25.0% increase in risk for previous incarceration. In Toronto and London, victimization was associated with increased risk for incarceration.

**Conclusion:**

This research highlights disproportionately high prevalence of ever being incarcerated among FNIM living in three Ontario cities. Experiencing racism, family disruption, and victimization are associated with incarceration. Decreasing the rates of family disruption, experiences of racism, and victimization should inform future policy and services to reduce the disproportionately high prevalence of incarceration for FNIM people living in urban settings.

## Résumé

**Objectifs:**

Les personnes autochtones présentent une prévalence démesurément élevée d’incarcération dans le système judiciaire canadien. Il y a cependant peu d’études dirigées par des Autochtones sur le colonialisme et le système judiciaire, en particulier sur les associations entre le racisme, les perturbations familiales imposées de l’extérieur et les antécédents d’incarcération. C’est pourquoi nous avons fait porter notre étude sur l’association entre la proportion d’incarcérations antérieures et de perturbations familiales, les expériences de racisme et la victimisation chez les adultes autochtones vivant à London, Thunder Bay et Toronto (Ontario), au Canada. Les trois villes ont dit ne pas vouloir que nous fassions de comparaisons entre elles; elles voulaient plutôt des analyses de leur ville pour savoir où des mesures de soutien supplémentaires étaient nécessaires.

**Méthode:**

Des partenaires associatifs autochtones ont utilisé l’échantillonnage en fonction des répondants (EFR) pour collecter des données auprès des personnes des Premières Nations, des Inuits et des Métis (PNIM) à London, Thunder Bay et Toronto. Les estimations de prévalence, les intervalles de confiance de 95 % et le risque relatif ont été présentés à l’aide de modèles de Poisson non pondérés et de proportions ajustées selon l’EFR.

**Résultats:**

La proportion de répondantes et de répondants ayant déjà été incarcérés était de 43 % à London, de 54 % à Toronto et de 72 % à Thunder Bay. Dans les trois villes, la fréquentation des services de protection de l’enfance et l’expérience du racisme étaient associées à une hausse d’environ 25 % du risque d’incarcération antérieure. À Toronto et à London, la victimisation était associée à un risque accru d’incarcération.

**Conclusion:**

Cette étude souligne la prévalence démesurément élevée de l’incarcération antérieure chez les personnes PNIM vivant dans trois villes de l’Ontario. L’expérience du racisme, les perturbations familiales et la victimisation étaient associées à l’incarcération. La réduction des taux de perturbations familiales, d’expérience du racisme et de victimisation devrait éclairer les politiques et les services futurs afin de réduire la prévalence démesurément élevée de l’incarcération chez les personnes PNIM vivant en milieu urbain.

## Introduction

There is an alarming gap in high-quality, comprehensive, and inclusive health, well-being, and social data for First Nations (Status and non-Status), Inuit, and Métis populations living in urban areas. Data from the Canadian government indicate that Indigenous peoples experience disproportionately high rates of incarceration in the Canadian justice system, and that the situation is worsening. Indigenous peoples make up approximately 5% of the Canadian population. However, since 2010, there has been a 52.1% increase among Indigenous peoples in the offender population compared to a 23.5% increase among non-Indigenous peoples (Office of the Correctional Investigator, 2019). Government data reports Indigenous incarceration in a given year. For example, in 2016–2017, 26.8% of offenders in Canada who were incarcerated were Indigenous (Office of the Correctional Investigator, 2019), while in 2017–2018 in Ontario, 12.2% of offenders in custody were Indigenous (Statistics Canada, 2020).

Colonialism and its associated impacts, including appropriation of land, resources, and the imposition of colonial societal structures, such as the legal and penal systems, have actively aimed to erode Indigenous sovereignty (Whitbeck et al., 2004; Truth and Reconciliation Commission, 2015). This has manifested in the disproportionately high risk of incarceration among Indigenous peoples. Further, disproportionately high rates of incarceration are likely to be underestimates as they rely on self-identification. It is well documented that misclassification and non-participation are common challenges in government data systems that rely on self-identification (see Smylie & Firestone, 2015). Indigenous peoples who offend are not likely to self-identify as self-identifying can have negative implications. An example of negative implications is that Indigenous peoples are more likely to finish their complete sentence and not get parole compared to non-Indigenous peoples. In 2015–2016, compared to 48% of non-Indigenous peoples, only 31% of Indigenous peoples were granted parole, and further, only 12% of Indigenous peoples had their cases prepared for a parole hearing when they were eligible for parole (Auditor General of Canada, 2016). This report only noted that offenders identified as Indigenous; it is not clear how this identification was specifically done. It is also possible for errors in self-identification to occur due to the purported benefits, e.g., non-Indigenous people’s misperception of reduced sentencing through Gladue courts. Thus, data available for Indigenous peoples in the justice system are at least somewhat unreliable.

Homel and colleagues (1999) noted that Indigenous involvement in the legal system should be viewed through a colonial lens which includes the context of the forced removal of Indigenous children from their families (e.g., residential schools, foster care), institutionalized racism, and victimization. Racism is particularly important to examine because it is not included in western theories of risk for justice involvement. For example, violence risk assessment tools that were developed on mainly white males are used with Indigenous people in the justice system and they do not include racism as a predictor of offending. Conversely, research does indicate that experiencing racism is associated with delinquent behaviour, aggression, and violence (Hartshorn et al., 2012; Mmari et al., 2010; Whitbeck et al., 2014).

Colonialism comprises past and ongoing direct and indirect violence (including physical violence) from settlers, government employees, the church, and police, and legislative violence such as the *Indian Act* which took and continues to remove First Nations’ human rights and continues to contribute to victimization (Brittain & Blackstock, 2015).

The Truth and Reconciliation Commission (2015) remarked that colonialism places Indigenous people at greater risk for incarceration because colonialism, which includes forced residential school attendance, causes a legacy of higher rates of addiction, mental health issues, family violence, parental incarceration, and child protection agency involvement. Approximately 150,000 First Nations, Inuit, and Métis children were forced into residential schools between the 1870s and 1997 (CBC, 2021). Further, few people recognize that this forced removal of children continues as there are currently three times the number of First Nations children in child welfare than there were at peak attendance levels of residential schools (First Nations Child and Family Caring Society, n.d.). As well, compared to non-Indigenous children, First Nations children are six to eight times more likely to be involved with child protection. According to 2016 Census data, 7.7% of children under the age of 14 years in Canada are Indigenous but 52.2% of children in foster care in Canada are Indigenous (Government of Canada, n.d.). There are no exact figures for Indigenous children who were adopted into non-Indigenous families from the 1960s to the early 1980s (the Sixties Scoop), but Indian Affairs recorded that 11,132 First Nations Status children were adopted out of their communities, although this is an underestimate due to the exclusion of Inuit or Métis children from these records (Erasmus & Dussault, 1996). There is evidence that for Indigenous peoples, foster care experiences are associated with incarceration (Cesaroni et al., 2019).

Colonial factors associated with legal system involvement include systematic discrimination and racism, family violence, family stress, victimization, intergenerational loss, trauma, substance use (as a means of coping with trauma), and exclusion from the education and economic systems which directly and indirectly impact employment and experiences of poverty (Brzozowski et al., 2006; La Prairie, 1992; Ferrante, 2012; Homel et al., 1999; Office of the Correctional Investigator, 2013). There is a dearth in the literature examining these factors for both Indigenous people with a history of incarceration *and* Indigenous people without a history of incarceration which is the purpose of the present study. Generally, research examining Indigenous peoples in the justice system actively ignores or is silent on the colonial context, commonly failing to incorporate the colonial context in the research question(s) or only briefly outlining these issues in the introduction. Further, very few justice studies actively include Indigenous communities in the research, nor do they include an Indigenous research team. Finally, there is paucity in research examining family disruption stemming from colonialism as a risk factor for incarceration.

Understanding incarceration pathways and experiences of colonialism among Indigenous peoples in urban settings is important as there is little comprehensive and reliable data on this population. This understanding of colonial policies and frameworks that link risk and protective factors to the longer-term impacts of justice involvement is key to generating policies and programs with longer-term impacts for Indigenous people living in urban settings. The objectives of this study were to examine the association between the rate of ever being incarcerated and family disruption, experiences of racism, and victimization for Indigenous adults in the cities of London, Thunder Bay, and Toronto, Ontario, Canada.

## Methods

To examine the proportions of incarceration and which factors are associated with incarceration, the three OHC projects used innovative research methods including Indigenous community-based partnerships, a respectful health survey developed in partnership with each community, and respondent-driven sampling (RDS) methods. The three study sites specifically asked that we did not statistically compare them as they wanted results that were specific to their community. Therefore, in the Results section, we highlight statistically significant results in each city separately.

### Community-based participatory research and recruitment

The three OHC sites of Toronto, London, and Thunder Bay included Indigenous community leadership in all aspects of study design, data collection/recruitment, analyses, and interpretation of data. Each community partner (Toronto: Seventh Generation Midwives of Toronto; London: Southwest Ontario Aboriginal Health Access Centre; Thunder Bay: Anishnawbe Mushkiki) retains full ownership and control over all data in accordance with the project’s community partnership data governance protocols and agreements with the Well Living House (WLH) which is consistent with United Nations Declaration on the Rights of Indigenous Peoples and Indigenous data sovereignty principles. Ethics approval for all sites was also obtained from Unity Health Toronto (#18–205, 09–108, 14–390). Each study used RDS to ensure valid and representative samples (see Rotondi et al., 2017) of FNIM community members in each city. Additional details for RDS recruitment procedures are included in Rotondi et al. (2017). The respectful health surveys were customized by each community and took approximately 90 min to complete.

### Study sites

In Toronto, ten seeds (recruiters) were initially selected as detailed by Rotondi et al. (2017). An additional ten seeds were used to increase recruitment speed later in the study. Initially, three coupons were issued per person, but this was later increased to five coupons to enhance the recruitment rate. For this study, all analyses are restricted to only those participants who resided in the city of Toronto at the time of the survey.

In London, data were collected between October 2015 and April 2016**.** Six seeds were selected. Participants were given three coupons upon completion of the interview with which to recruit family or friends (see Firestone et al., 2018).

For OHC Thunder Bay, data were collected between March and August 2019. Twelve seeds were selected, and three coupons were given to each participant. Upon completing their interview, participants were given three coupons to recruit family or friends.

### Outcome

For the three study sites, justice system involvement questions included the following: “*Have you ever done time in jail?*” (“yes” or “no”); and “*If yes, was this for a federal or provincial offense/crime?*” For Thunder Bay, the community decided to only ask those who had been in jail for 96 h or more whether this was for a federal and/or provincial offense/crime. Provincial incarceration denotes being incarcerated for 2 years less a day and the crimes committed to be provincially incarcerated are less serious, whereas federal incarceration is for more serious crimes and denotes being incarcerated for more than 2 years.

### Explanatory variables

All variables were obtained from the OHC surveys in their respective sites. There were some minor variations in how residential school attendance was coded between the three cities. For Toronto, there was no “unsure” category, so “no” was always coded as “no,” whereas for London and Thunder Bay, categories of “unsure” and “don’t know/decline” were combined. Thus, two regression models are presented: (1) a three-category model including the “unsure/don’t know/decline” variable and (2) a conservative model where the “unsure/don’t know/decline” are considered “no.” For the regressions, racism was defined as “ever been treated poorly or unfairly because you are Indigenous” and was dichotomous (yes/no).

All variables, except for the victimization scale (which is continuous), are dichotomous. Indigenous identity was determined by asking participants how they self-identified. For household income, before-tax low-income cut-off (LICO) was used. LICO was determined using reported household income, reported number of individuals living off reported household income, and the overall population size of each site. Employment included employed (part-time, full-time, seasonal, self-employed, homemaker, informal paid work), unemployed, and not in labour force. For household type, no regular dedicated physical accommodation indicated homeless or living in a shelter. Participants from all three cities with children were asked whether they felt they had enough time with their child. Education included some high school or less, completed high school, some or completed college, and some or completed university. Ceremony and traditional medicine use were also explored to help elicit and understand their intrinsic value and benefits. These variables were chosen because research indicates that these are risk factors for justice involvement and we hypothesized that there would be differences between the two groups (see Andrews et al., 2006).

The victimization experiences scale included being physically hurt, insulted, threatened, and screamed at, having actions restricted, and being forced to have non-consensual sex. The questions ask if anyone in the participant’s household has *ever* harmed them. The total score of the victimization scale was included with a range consisting of 0 to 6 (0 indicating that no victimization was experienced). Each variable on the victimization scale was also examined separately.

### Statistical analysis

Seeds were excluded in all analyses. Descriptive statistics (Tables 1, 2, and 3) were produced in RStudio using RDS-II estimates and the RDS package in R. Statistical significance between the groups who have experienced incarceration vs. those who have not was determined using the MOVER method (Rotondi, 2014) for each site. The MOVER method was used as it allows construction of risk differences, a limitation of traditional RDS analyses which can only estimate proportions of interest and has greater power to detect differences compared to the overlap method (Rotondi, 2014).

**Table 1:** Our Health Counts Toronto weighted frequency estimates

	Toronto incarceration history % (95% CI), *n* = 481	Toronto no incarceration history % (95% CI), *n* = 417	Toronto total % (95% CI), *n* = 906*	MOVER results difference in proportions, % (95% CI)
Indigenous identity				
First Nations	86.7 (81.2, 92.2)	84.4 (78.6, 90.2)	85.6 (81.7, 89.5)	2.3 (− 5.7, 10.3)
First Nations and Métis	0.8 (0.0, 2.4)	0.1 (0.0, 0.3)	0.4 (0.3, 1.2)	0.7 (− 0.2, 2.3)
Inuit	0.2 (0.0, 0.8)	0.6 (0.0, 1.4)	0.4 (0.0, 0.9)	− 0.4 (− 1.3, 0.4)
Métis	11.8 (6.5, 17.1)	14.9 (9.2, 20.7)	13.2 (9.4, 17.0)	− 3.1 (− 10.9, 4.7)
Other	0.5 (0.0, 1.1)	-	0.3 (0.0, 0.5)	-
Age in years				
15 to 24	5.1 (1.0, 9.1)	39.9 (32.1, 47.6)	21.4 (16.7, 26.0)	** − 34.8** (**− **43.5, − 26.1)
25 to 34	21.0 (14.5, 27.6)	18.5 (12.0, 25.0)	19.5 (14.8, 24.1)	2.5 (**− **6.8, 11.8)
35 to 44	25.4 (19.1, 31.7)	15.9 (9.9, 22.00)	21.6 (17.0, 26.2)	9.5 (**− **.7, 18.)
45 to 54	34.4 (27.1, 41.8)	11.7 (7.0, 16.3)	23.6 (19.0, 28.2)	**22.8** (10.1, 31.4)
55 to 64	11.9 (6.8, 17.0)	9.3 (3.7, 14.9)	10.6 (6.9, 14.2)	2.63 (**− **4.9, 10.2)
65 and over	2.2 (0.0, 6.0)	4.8 (0.8, 8.8)	3.4 (0.6, 6.2)	** − **2.6 (**− **7.2, 3.0)
Gender				
Female	33.4 (26.5, 40.3)	65.0 (57.5, 72.5)	48.1 (42.4, 53.8)	** − 31.6** (**− **41.8, − 21.4)
Male	65.3 (58.3, 72.3)	33.2 (25.9, 40.6)	50.4 (44.7, 56.1)	**32.1** (22.0, 42.3)
Other	0.1 (0.0, 0.3)	1.0 (0.0, 3.1)	0.5 (0.0, 1.5)	** − **0.9 (**− **3.0, 0.1)
Transgendered	1.2 (0.0, 2.6)	0.8 (0.6, 1.0)	1.0 (0.3, 1.7)	0.4 (**− **0.8, 1.8)
Education				
Some high school or less	49.4 (41.5, 57.3)	48.9 (40.8, 57.1)	49.5 (43.8, 55.2)	0.4 (− 10.9, 11.8)
Completed high school	19.8 (12.8, 26.7)	15.0 (9.0, 20.9)	17.7 (13.0, 22.4)	4.8 (− 4.4, 14.0)
Some or completed college	25.9 (19.7, 32.1)	24.7 (17.6, 31.8)	24.9 (20.2, 29.6)	1.2 (− 8.2, 10.6)
Some or completed university	5.0 (1.8, 8.1)	10.7 (5.6, 15.8)	7.6 (4.6, 10.6)	− 5.7 (− 11.8, 0.3)
Chose not to answer	-	0.7 (0.0, 2.9)	0.4 (0.0, 1.4)	-
LICO (below cut-off)	89.1 (84.5, 93.7)	84.1 (77.7, 90.5)	86.9 (83.0, 90.8)	5.0 (− 2.9, 12.8)
Employment				
Employed	16.1 (10.2, 21.9)	19.4 (12.5, 26.3)	17.3 (12.8, 21.8)	− 3.3 (− 12.4, 5.8)
Unemployed	76.6 (69.6, 83.5)	43.9 (35.9, 52.0)	61.6 (56.1, 67.2)	**32.6 (22.0, 43.3)**
Not in labour force	4.4 (0.4, 8.4)	34.2 (26.8, 41.7)	18.4 (13.9, 22.8)	− **29.8 (**− **39.3,** − **21.4)**
Chose not to answer	2.9 (0.3, 5.6)	2.5 (0.7, 4.3)	2.7 (1.1, 4.3)	0.5 (− 2.8, 3.7)
Household type				
Housing with adequate/sufficient physical conditions	35.9 (28.2, 43.6)	60.6 (52.9, 68.4)	47.4 (41.71 53.2)	** − 24.8 (− 35.7, − 13.9)**
Housing with inadequate/insufficient physical conditions	15.3 (9.0, 21.6)	15.9 (10.1, 21.6)	15.3 (11.0, 19.5)	** − **0.6 (**− **9.1, 7.9)
No regular dedicated physical accommodation	36.7 (29.7, 43.7)	14.7 (10.3, 19.1)	26.9 (22.4, 31.5)	**22.0 (13.7, 30.3)**
Unclear housing conditions	12.2 (7.1, 17.3)	8.8 (4.4, 13.2)	10.4 (7.1, 13.7)	3.4 (**− **3.4, 10.2)
Food security				
Often not enough to eat	6.7 (2.5, 10.9)	6.7 (2.4, 11.0)	6.7 (3.7, 9.8)	0.0 (− 6.0, 6.0)
Sometimes not enough to eat	22.2 (15.0, 29.4)	15.6 (10.0, 21.7)	18.7 (14.0, 23.3)	6.6 (− 2.9, 15.8)
Always enough of kinds of food	16.5 (11.4, 21.6)	23.4 (16.5, 30.4)	19.8 (15.5, 24.1)	− 7.0 (− 15.6, 1.7)
Enough food, not of kind you wanted	53.7 (45.9, 61.6)	54.3 (46.2, 62.4)	54.2 (48.5, 59.9)	− 0.6 (− 11.9, 10.7)
Residential school				
Residential school attendance	11.7 (6.5, 16.8)	9.5 (6.2, 12.8)	10.4 (7.3, 13.5)	2.2 (− 4.0, 8.3)
Family member residential school attendance	63.2 (55.6, 70.7)	52.9 (44.7, 61.1)	58.6 (53.0, 64.3)	10.3 (− 0.9, 21.4)
Sixties Scoop	25.7 (18.7, 32.7)	22.0 (15.0, 28.9)	24.8 (19.6, 30.0)	3.8 (− 6.1, 13.7)
CPA involvement as a child	51.9 (44.0, 59.7)	38.8 (30.8, 46.8)	46.2 (40.5, 51.9)	**13.1 (1.9, 24.3)**
Family member/close friend gone missing	25.6 (19.2, 32.1)	26.0 (19.1, 32.8)	25.7 (21.0, 30.3)	− 0.3 (− 9.7, 9.1)
Family member/close friend died from violence	42.6 (34.8, 50.3)	25.5 (18.1, 33.0)	33.7 (28.4, 39.0)	**17.0 (6.3, 27.8**)
Racism ever	60.1 (52.2, 68.1)	45.3 (37.2, 53.4)	53.7 (47.9, 59.5)	**14.8 (3.5, 26.2)**
Racism past 12 months	54.2 (44.1, 64.3)	59.9 (49.7, 70.1)	57.8 (50.8, 64.8)	− 5.6 (− 20.0, 8.7)
Participated in ceremony	68.4 (60.6, 76.3)	59.3 (51.0, 67.6)	64.5 (58.8, 70.3)	9.2 (− 2.2, 20.6)
Used traditional medicines	44.9 (37.2, 52.7)	51.6 (43.4, 59.8)	48.9 (43.2, 54.6)	− 6.7 (− 18.0, 4.6)
Adequate Toronto resources for				
Legal services	43.2 (35.4, 51.0)	40.4 (32.5, 48.4)	41.1 (35.5, 46.7)	2.8 (− 8.3, 13.9)
Dealing with incarceration	26.9 (19.9, 33.9)	21.8 (15.7, 27.9)	24.0 (19.3, 28.7)	5.1 (− 4.2, 14.4)
Children under 18	30.1 (23.0, 37.1)	22.6 (16.1, 29.2)	27.2 (22.2, 32.2)	7.4 (− 2.2, 17.0)
If have child				
Plenty or enough time spent with child	18.5 (9.1, 27.9)	65.8 (52.9, 78.8)	36.3 (26.80, 45.9)	− **47.3 (**− **63.4,** − **31.3)**
Wish could spend more time with child	81.5 (72.1, 90.9)	34.2 (21.2, 47.1)	63.7 (54.2, 73.2)	**47.3 (31.3, 63.4)**
	***M* (95% CI)**	***M* (95% CI)**	***M* (95% CI)**	MOVER risk difference in means** (95% CI)
Victimization experiences	2.6 (2.3, 3.0)	2.1 (1.8, 2.4)	2.3 (2.1, 2.6)	**0.5 (0.0, 1.0)**

Variables with less than six individuals in a cell were not reported. The * indicates that there are missing data on the incarceration variable. *M,* mean; *95% CI,* confidence interval. Bold text indicates significant difference between groups. The ** indicates that this is coded as the means for the victimization scale

**Table 2:** Our Health Counts London weighted frequency estimates

	London incarceration history % (95% CI), *n* = 238	London no incarceration history % (95% CI), *n* = 244	London total % (95% CI), *n* = 484*	MOVER risk difference in proportions, % (95% CI)
Indigenous identity				
First Nations	99.5 (98.5, 100.0)	90.8 (85.1, 96.5)	94.6 (91.3, 98.0)	**8.7 (2.9, 14.5)**
First Nations and Métis	0.3 (0.0, 1.4)	-	0.1 (0.0, 0.4)	-
Inuit	-	3.5 (2.8, 4.1)	2.0 (1.7, 2.2)	-
Métis	0.1 (0.0, 0.4)	5.8 (0.0, 11.5)	3.3 (0.0, 6.6)	− 5.6 (− 11.3, 0.1)
Age (years)				
15 to 24	17.9 (9.4, 25.6)	43.1 (31.6, 54.7)	31.9 (23.9, 39.9)	− **25.7 (**− **69.4,** − **11.5)**
25 to 34	16.4 (6.7, 26.1)	26.8 (16.2, 37.3)	22.2 (15.0, 29.3)	− 10.3 (− 24.7, 4.0)
35 to 44	17.0 (10.0, 24.1)	15.6 (8.3, 23.0)	16.6 (11.4, 21.71)	1.4 (− 8.8, 11.6)
45 to 54	22.8 (12.7, 32.9)	8.5 (4.2, 12.8)	14.7 (9.4, 20.0)	**14.4 (3.4, 25.3)**
55 to 64	14.3 (5.7, 22.8)	4.4 (0.8, 7.8)	8.7 (4.1, 13.2)	**9.9 (0.7, 19.2)**
65 and over	11.7 (4.8, 18.6)	1.6 (0.0, 3.4)	6.0 (2.6, 9.3)	**10.2 (3.1, 17.3)**
Gender				
Female	26.0 (16.2, 35.8)	60.0 (48.5, 71.6)	45.5 (37.4, 53.5)	** − 34.0 (− 49.2, − 18.9)**
Male	74.0 (64.2, 83.8)	38.6 (27.0, 50.2)	53.7 (45.7, 61.8)	**35.4 (20.2, 50.6)**
Other	-	0.0 (0.0, 0.0)	0.0 (0., 0.01)	-
Transgendered	-	1.9 (1.0, 1.8)	0.8 (0.6, 0.9)	-
Education				
Some high school or less	63.0 (52.3, 73.8)	38.2 (27.1, 49.4)	49.2 (41.1, 57.2)	**24.8 (9.3, 40.3)**
Completed high school	14.4 (5.9, 22.9)	25.9 (15.7, 36.2)	20.8 (14.1, 27.6)	− 11.5 (− 24.8, 1.8)
Some or completed college	17.9 (11.6, 24.1)	27.1 (17.1, 37.1)	23.0 (16.7, 29.4)	− 9.2 (− 21.0, 2.6)
Some or completed university	4.7 (0.0, 11.6)	8.8 (4.4, 13.1)	7.0 (2.8, 11.1)	− 4.1 (− 10.5, 4.1)
LICO (below cut-off)	89.1 (80.6, 97.6)	90.9 (84.5, 97.3)	90.1 (84.8, 95.3)	− 1.8 (− 12.5, 8.8)
Employment				
Employed	21.7 (12.3, 31.0)	25.0 (15.0, 35.1)	23.5 (16.5, 30.5)	− 3.4 (− 17.1, 10.3)
Unemployed	61.4 (50.2, 72.6)	51.5 (39.8, 63.2)	55.6 (47.3, 63.8)	9.8 (− 6.4, 26.0)
Not in labour force	15.0 (7.1, 22.9)	22.7 (11.8, 33.7)	19.3 (12.3, 26.3)	− 7.7 (− 21.3, 5.8)
Chose not to answer	1.2 (0.0, 6.2)	0.7 (0.0, 1.7)	1.7 (0.0, 3.5)	0.5 (− 1.1, 5.6)
Household type				
Housing with adequate/sufficient physical conditions	55.0 (43.8, 66.2)	68.9 (58.8, 79.0)	63.0 (55.4, 70.6)	− 13.9 (− 29.0, 1.2)
Housing with inadequate/insufficient physical conditions	16.2 (8.8, 23.6)	13.8 (6.6, 20.9)	14.8 (9.6, 19.9)	2.5 (− 7.9, 12.8)
No regular dedicated physical accommodation	22.1 (13.2, 31.0)	9.0 (2.4, 15.6)	14.6 (9.1, 20.2)	**13.1 (2.0, 24.2)**
Unclear housing conditions	6.7 (1.0, 12.5)	8.4 (3.1, 13.7)	7.6 (3.7, 11.5)	− 1.7 (− 9.5, 6.2)
Food security				
Often not enough to eat	6.7 (0.0, 13.9)	5.6 (1.3, 9.9)	6.6 (2.0, 10.2)	1.1 (− 6., 9.5)
Sometimes not enough to eat	17.4 (10.1, 24.6)	14.5 (7.2, 21.8)	15.7 (10.6, 20.8)	2.9 (− 7.4, 13.2)
Always enough of kinds of food	24.5 (13.8, 35.3)	19.2 (10.0, 28.4)	21.4 (14.2, 28.9)	5.4 (− 8.8, 19.5)
Enough food, not of kind you wanted	51.4 (40.0, 92.9)	60.7 (49.7, 71.7)	56.0 (48.8, 64.9)	− 9.3 (− 25.2, 33.6)
Residential school				
Residential school attendance	4.5 (0.3, 8.6)	2.4 (0.0, 5.2)	3.3 (0.8, 5.7)	2.1 (− 3.0, 6.9)
Family member residential school attendance	65.5 (54.0, 77.0)	70.2 (59.6, 80.8)	68.3 (60.3, 76.2)	− 4.7 (− 20.3, 11.0)
Sixties Scoop	15.8 (8.0, 23.6)	17.4 (10.5, 24.3)	16.7 (11.5, 21.8)	− 2.0 (− 12.0, 8.8)
CPA involvement as a child	48.3 (37.1, 59.4)	37.0 (25.6, 48.5)	42.1 (34.1, 50.2)	11.3 (− 4.7, 27.3)
Family member/close friend gone missing	24.9 (16.0, 33.7)	31.3 (21.0, 41.5)	284 (21.4, 35.3)	− 6.4 (− 20.0, 7.1)
Family member/close friend died from violence	38.9 (27.7, 50.2)	29.7 (20.8, 38.7)	33.6 (26.3, 40.8)	9.2 (− 5.2, 23.6)
Racism ever	68.2 (56.9, 79.6)	56.6 (45.2, 68.0)	61.4 (53.3, 69.6)	11.7 (− 4.5, 27.8)
Racism past 12 months	45.2 (34.4, 56.1)	32.2 (22.0, 42.4)	37.7 (30.3, 45.1)	13.1 (− 1.8, 28.0)
Participated in ceremony	64.4 (53.54, 75.2)	66.6 (55.2, 77.9)	65.7 (57.9, 73.6)	− 2.2 (− 17.9, 13.5)
Used traditional medicines	54.7 (43.4, 66.1)	69.0 (59.0, 79.0)	63.0 (55.2, 70.7)	− 14.2 (− 29.4, 0.9)
Adequate London resources for				
Legal services	52.5 (39.8, 65.1)	48.7 (36.3, 61.0)	50.5 (41.5, 59.5)	3.8 (− 13.9, 21.5)
Dealing with incarceration	30.5 (19.3, 41.8)	41.9 (28.9, 54.9)	36.9 (28.0, 45.7)	− 11.4 (− 28.5, 5.8)
Children under 18	37.0 (25.5, 48.5)	40.7 (29.7, 51.7)	39.3 (31.3, 47.3)	− 3.7 (− 19.6, 12.2)
If have child				
Plenty or enough time spent with child	33.0 (17.7, 48.3)	53.8 (37.2, 70.4)	45.9 (34.0, 57.8)	− 20.8 (− 43.4, 1.8)
Wish could spend more time with child	67.0 (51.7, 82.3)	46.2 (29.6, 62.8)	54.1 (42.2, 66.0)	20.8 (− 1.8, 43.4)
	**M (95% CI)**	**M (95% CI)**	**M (95% CI)**	MOVER risk difference in means** (95% CI)
Victimization ever experiences	2.3 (1.9, 2.6)	2.2 (1.7, 2.7)	2.3 (1.9, 2.6)	0.0 (− 0.6, 0.7)

Variables with less than six individuals in a cell were not reported. The * indicates that there are missing data on the incarceration variable. *M,* mean; *95% CI*, confidence interval. Bold text indicates significant difference between groups. The ** indicates that this is coded as the means for the victimization scale

**Table 3:** Our Health Counts Thunder Bay weighted frequency estimates

		Thunder Bay incarceration history % (95% CI), *n* = 306	Thunder Bay no incarceration history % (95% CI), *n* = 119	Thunder Bay total % (95% CI), *n* = 589*		MOVER difference in proportions, % (95% CI)
Indigenous identity						
First Nations		96.9 (93.2, 100.0)	98.5 (95.6, 100.0)	97.9 (95.7, 100.0)		− 1.6 (− 5.6, 2.7)
First Nations and Métis		0.9 (0.0, 2.8)	-	0.6 (0.0, 1.7)		-
Inuit		-	-	-		-
Métis		2.2 (0.0, 5.4)	1.5 (0.0, 4.4)	1.5 (0.0, 3.4)		0.7 (− 3.0, 4.2)
Age in years						
15 to 24		12.8 (6.7, 18.9)	20.5 (6.6, 34.5)	17.3 (12.3, 22.4)		− 7.8 (− 23.0, 7.5)
25 to 34		32.1 (24.2, 40.0)	33.0 (18.4, 47.5)	30.1 (24.3, 35.8)		− 0.9 (− 17.4, 15.7)
35 to 44		18.0 (10.8, 25.3)	30.1 (16.5, 43.4)	20.9 (15.5, 26.2)		− 11.9 (− 27.2, 3.3)
45 to 54		22.0 (13.9, 30.1)	4.67 (0.7, 8.6)	15.9 (10.9, 20.9)		**17.4 (8.3, 26.4)**
55 to 64		14.8 (8.2, 21.4)	11.8 (4.2, 19.4)	13.2 (8.9, 17.5)		3.0 (− 7.0, 13.1)
65 and over		0.3 (0.0, 0.5)	0.1 (0.0, 0.4)	2.6 (0.6, 4.7)		0.2 (− 0.2, 0.4)
Gender						
Female		32.3 (23.7, 40.9)	50.0 (34.8, 65.2)	47.3 (40.8, 53.8)		** − 17.7 (− 35.2, − 0.2)**
Male		67.7 (59.1, 76.3)	50.0 (34.8, 65.3)	52.7 (46.2, 59.2)		**17.7 (0.2, 35.2)**
Other		-	-	-		-
Transgendered		-	-	-		-
Education						
Some high school or less		66.7 (58.3, 75.0)	85.5 (75.3, 95.7)	68.6 (62.8, 74.4)		** − 18.9 (− 32.1, − 5.7)**
Completed high school		12.6 (6.2, 19.0)	7.4 (0.0, 16.1)	15.3 (10.8, 19.8)		5.2 (**− **5.6, 15.0)
Some or completed college		16.0 (10.1, 22.0)	4.1 (0.0, 8.4)	12.0 (8.3, 15.8)		**12.0 (4.6, 19.2)**
Some or completed university		4.7 (1.0, 8.5)	3.0 (0.0, 7.5)	4.1 (1.6, 6.6)		1.7 (**− **4.2, 6.5)
LICO (below LICO cut-off)		90.7 (84.5, 96.9)	90.3 (80.1, 100.0)	87.4 (82.8, 91.9)		0.4 (**− **11.1, 12.3)
Employment						
Employed		17.3 (10.9, 23.7)	7.3 (0.5, 14.0)	15.1 (10.5, 19.7)		**10.0 (0.7, 19.3)**
Unemployed		64.2 (55.8, 72.5)	82.6 (72.2, 93.1)	67.3 (61.4, 73.3)		** − 18.5 (− 31.8, − 5.1)**
Not in labour force		18.6 (11.9, 25.2)	10.1 (1.6, 18.5)	17.6 (13.0, 22.2)		8.5 (**− **2.3, 19.2)
Household type						
Housing with adequate/sufficient physical conditions		50.2 (41.2, 59.2)	39.1 (24.5, 53.7)	47.9 (41.4, 54.4)		11.1 (− 6.1, 28.3)
Housing with inadequate/insufficient physical conditions		19.6 (12.1, 27.2)	32.1 (19.8, 44.3)	25.2 (19.9, 30.5)		− 12.4 (− 26.8, 2.0)
No regular dedicated physical accommodation		28.5 (20.2, 36.3)	26.1 (11.1, 41.2)	24.6 (18.7, 30.4)		2.4 (− 14.8, 19.3)
Unclear housing conditions		2.0 (0.0, 4.2)	2.7 (0.0, 8.7)	2.4 (0.4, 4.4)		− 0.8 (− 7.0, 2.7)
Food security						
Often not enough to eat		11.9 (6.0, 17.8)	18.9 (3.9, 33.9)	12.0 (7.4, 16.6)		− 7.0 (− 23.1, 9.1)
Sometimes not enough to eat		15.4 (7.0, 23.74	11.4 (7.0, 15.9)	16.1 (11.0, 21.2)		3.9 (− 5.6. 13.4)
Always enough of kinds of food		19.6 (12.8, 26.3)	20.1 (7.6, 32.6)	18.1 (13.3, 22.9)		− 0.5 (− 14.7, 13.7)
Enough food, not of kind you wanted		53.1 (43.6, 62.5)	49.6 (33.9, 65.3)	53.6 (46.9, 60.2)		3.5 (− 14.9, 21.9)
Residential school						
Residential school attendance		21.4 (13.6, 29.2)	13.6 (3.8, 23.4)	18.8 (13.6, 24.0)		7.8 (− 4.8, 20.3)
Family member residential school attendance		80.2 (72.3, 88.0)	72.5 (58.4, 86.7)	77.1 (71.1, 83.0)		7.6 (− 8.6, 23.8)
Sixties Scoop		21.4 (14.4, 28.5)	17.9 (5.3, 30.5)	19.6 (14.3, 24.8)		3.5 (− 11.0, 18.0)
CPA involvement as a child		52.7 (43.7, 61.8)	64.8 (50.4, 79.2)	50.0 (43.5, 56.5)		− 12.1 (− 29.2, 5.0)
Family member/close friend gone missing		38.3 (29.6, 47.0)	15.5 (3.5, 27.4)	31.1 (25.0, 37.2)		**22.8 (8.1, 37.6)**
Family member/close friend died from violence		55.9 (46.9, 64.9)	48.1 (33.1, 63.2)	49.1 (42.6, 55.6)		7.8 (− 9.7, 25.4)
Racism ever		68.4 (60.0, 76.9)	60.3 (45.7, 74.9)	66.3 (59.8, 72.7)		8.1 (− 8.7, 25.0)
Racism past 12 months		72.1 (63.7, 80.5)	66.0 (49.8, 82.2)	71.4 (65.0, 77.7)		6.1 (− 12.1, 24.4)
Participated in ceremony		59.2 (50.2, 68.2)	40.0 (25.1, 55.0)	52.9 (46.3, 59.5)		**19.2 (1.7, 36.6)**
Used traditional medicines		41.1 (32.4, 49.7)	24.3 (11.3, 37.3)	39.7 (33.4, 46.1)		**16.8 (1.2, 32.4)**
Adequate Thunder Bay resources for						
Legal services		56.5 (47.3, 65.6)	67.4 (52.7, 82.1)	53.3 (46.7, 59.9)		− 10.9 (− 28.3, 6.4)
Dealing with incarceration		22.9 (15.5, 30.4)	21.9 (11.2, 32.6)	21.4 (16.2, 26.6)		1.1 (− 12.0, 14.1)
Children under 18		58.2 (49.3, 67.1)	38.1 (22.6, 53.7)	49.3 (42.7, 55.9)	**20.1 (2.2, 38.0)**	
If have child						
Plenty or enough time spent with child		36.2 (23.9, 48.6)	47.3 (28.5, 66.1)	42.3 (33.1, 51.4)	− 11.10 (− 33.6, 11.4)	
Wish could spend more time with child		63.8 (51.5, 76.1)	48.0 (28.0, 67.49)	57.0 (47.9, 66.2)	15.8 (− 7.3, 38.8)	
	**M (95% CI)**		**M (95% CI)**	**M (95% CI)**		MOVER risk difference in means** (95% CI)
Victimization ever experiences	2.6 (2.2, 3.0)		1.6 (0.9, 2.3)	2.3 (2.0, 2.6)		**1.0 (0.2, 1.8)**

Variables with less than six individuals in a cell were not reported. The * indicates that there are missing data on the incarceration variable. *M,* mean; *95% CI,* confidence interval. Bold text indicates significant difference between groups. The ** indicates that this is coded as the means for the victimization scale

Multiple regression analyses were used to adjust for potential confounding effects of age, gender, and education level. Unweighted Poisson regression (Avery et al., 2019) with robust estimators of the variance was used for each OHC database since it estimates relative risks (RR) and maintains the nominal type 1 error rate for RDS regression models. For relative risk, an RR greater than 1 implies that the variable increases the risk of having ever done time in jail. Case deletion was used to account for missingness.

#### Missing data

There were some participants with missing data (< 1%) on the primary outcome, “*Have you ever done time in jail*?” All other variables including Indigenous identity, age, gender, education, income, and type of household had no data missing for Toronto, < 3% missing for London (only income), and < 5% missing for Thunder Bay. Given the small amount of missingness, we used case deletion. There were large amounts of missing data for the victimization scale. Proportionate to the sample size, Thunder Bay had the most missingness with 54.0% versus 31.0% and 43.0% for London and Toronto, respectively.

## Results

To simplify reporting, all described results are statistically significant, while complete results are presented in Tables 1, 2, and 3.

### OHC Toronto

#### History of incarceration

In Toronto, 54% of participants had a history of incarceration. There were significantly more 45- to 54-year-olds in the incarceration history group (34.4% versus 11.7%) and significantly more males (65.3% versus 33.2%) relative to the non-incarcerated group, respectively (Table 1). Compared to non-incarcerated participants, there were significantly more individuals who were unemployed (76.6% versus 43.9%) in the history of incarceration group. The incarceration history group also had a significantly higher proportion of people experiencing homelessness (36.7% versus 14.7%).

Significantly more individuals with a history of incarceration had child protection agency (CPA) involvement as children (51.9% versus 38.8% of non-incarceration history individuals). The incarceration history group had significantly more family members or close friends die from violence (42.6% versus 25.5%) and experienced significantly more anti-Indigenous racism as well (60.1% versus 45.3% for the non-incarcerated group).

Finally, the group with a history of incarceration reported a significantly higher victimization scale mean (2.61) compared to those with no history of incarceration (*M* = 2.13). For the history of incarceration group (*n* = 468), 90.1% had been provincially incarcerated and 9.8% had been federally incarcerated.

#### No history of incarceration

There were significantly more youth (aged 15 to 24 years) in the non-incarcerated group compared to the history of incarceration group (39.9% and 5.1% respectively). The non-incarcerated group had significantly more females than the incarceration history group (65.0% versus 33.4%) and included more people not in the labour force (e.g., retired or student; 34.2% non-incarceration versus 4.4% in the history of incarceration group). The non-incarcerated group had a significantly higher proportion of adequate housing (60.6%) compared to the history of incarceration group (35.9%). Finally, for individuals with children, the non-incarcerated group reported having plenty or enough time spent with children significantly more (65.8%) than did the history of incarceration group (18.5%).

### OHC London

#### History of incarceration

In London, 49.0% of participants had a history of incarceration (see Table 2). Compared to the group with no history of incarceration (*n* = 244), the group with a history of incarceration (*n* = 238) had significantly more First Nations individuals (99.5% versus 90.8%). There were also significantly more individuals with a history of incarceration who were 45 to 54 years old (22.8% versus 8.5% no history of incarceration), 55 to 64 years old (14.3% history of incarceration versus 4.4%), and over the age of 65 (11.7% history of incarceration versus 1.6%). There were significantly more males in the incarceration history group (74.0% versus 38.6%). The incarceration history group had significantly less education with 63.0% having some high school or less and 38.2% of the non-incarcerated group having some high school or less. Compared to adults with no history of incarceration, significantly more adults with a history of incarceration were experiencing homelessness (22.1% versus 9.0%). For the history of incarceration group (*n* = 229), 81.5% had been provincially incarcerated and 18.5% had been federally incarcerated.

#### No history of incarceration

In London, there were significantly more youth aged 15 to 24 years in the group with no history of incarceration (43.1%) compared to the history of incarceration group (17.5%). Compared to the individuals with a history of incarceration, there were significantly more females in the non-incarcerated group (60.0% versus 26.0%).

### OHC Thunder Bay

#### History of incarceration

Thunder Bay had the highest proportion of history of incarceration with 72.0% of participants having a history of incarceration (see Table 3). Compared to the group with no history of incarceration (*n* = 119), the group with a history of incarceration (*n* = 306) had significantly more participants (22.0%) in the 45- to 54-year-old group compared to the non-incarcerated group (*n* = 119). There were significantly more males in the incarceration history group (50.0% versus 32.3%). The incarceration history group had a significantly higher proportion of some or completed college (16.0% versus 4.1% of the group with no history of incarceration). There were significantly more employed adults in the history of incarceration group (17.3%) than the group with no incarceration history (7.3%). The history of incarceration group had significantly more members who had a family member or close friend go missing (38.3% versus 15.5%). As well, the history of incarceration group had a significantly higher average on the victimization scale (2.6) compared to 1.6 for the non-incarceration group. In terms of strengths, the adults with a history of incarceration had a significantly higher proportion of participating in ceremony (59.2% versus 40.0%) and using traditional medicines (41.1% versus 24.3% of non-incarcerated participants).

Finally, compared to the group with no incarceration history, those with a history of incarceration were significantly more likely to have children under the age of 18 (63.8% versus 48.0%). For the history of incarceration sample (*n* = 272), 88.3% had been provincially incarcerated, 6.7% had been federally incarcerated, and 4.4% have been both provincially and federally incarcerated.

#### No history of incarceration

Compared to the adults with a history of incarceration, the Thunder Bay adults with no incarceration history had significantly more females (50.0% versus 32.3%). The adults with no history of incarceration also had significantly less education (85.5% had some high school or less) than their peers with a history of incarceration (66.7%). Further, the non-incarceration group had a significantly higher proportion of participants who were unemployed (82.6% versus 64.2%).

### Regression models

For each *OHC* study location, separate Poisson regression models were used to examine factors associated with ever being incarcerated. In each of the Toronto, London, and Thunder Bay locations, the adjusted regression models showed that having a history of CPA involvement was associated with a higher risk of ever being incarcerated. Moreover, these effects were large as point estimates suggest that child protection agency involvement leads to an approximately 25.0% increase in the risk of ever being incarcerated. Similarly, adjusted Poisson regression models showed that ever experiencing racism because they were Indigenous led to a statistically significant increase in the probability of ever having been incarcerated in each *OHC* study location (see Table 4).

**Table 4:** Poisson regression models examining factors associated with the risk of ever being incarcerated

	OHC Toronto *N* = 906		OHC London *N* = 484		OHC Thunder Bay *N* = 589	
	Crude RR (95% CI) [*N*]	Adjusted RR* (95% CI) [*N*]	Crude RR (95% CI) [*N*]	Adjusted* RR (95% CI) [*N*]	Crude RR (95% CI) [*N*]	Adjusted RR (95% CI) [*N*]
Any CPA involvement	**1.22** **(1.08, 1.38)** **[891]**	**1.27** **(1.14, 1.42)** **[889]**	1.19 (0.99, 1.42) [479]	**1.30** **(1.11, 1.53)** **[477]**	**1.18** **(1.01, 1.38)** **[555]**	**1.25** **(1.07, 1.46)** **[552]**
Residential school Self	1.16 (0.96, 1.41) [891]	1.04 (0.87, 1.26) [889]	**1.41** **(1.04, 1.90)** **[479]**	1.28 (0.96, 1.71) [477]	0.92 (0.75,1.14) [534]	0.89 (0.72, 1.10) [531]
Residential school Other family – unsure as its own	1.01 (0.87, 1.16) [898]	1.10 (0.95, 1.25) [896]	Yes: 0.87 (0.69, 1.09) Unsure: 0.93 (0.65, 1.31 [482]	Yes: 0.99 (0.80, 1.22) Unsure: 1.00 (0.72, 1.38) [480]	Yes: 1.04 (0.77, 1.42) Unsure: 1.23 (0.85, 1.79) [560]	Yes: 1.17 (0.86, 1.59) Unsure: 1.24 (0.86, 1.79) [557]
Residential school Other family – conservative (unsure/don’t know = no)	-	-	0.89 (0.73, 1.09) [482]	0.99 (0.83, 1.18) [480]	0.93 (0.76, 1.14) [560]	1.04 (0.86, 1.27) [557]
Racism	**1.28** **(1.12, 1.47)** **[892]**	**1.35** **(1.19, 1.54)** **[890]**	1.18 (0.94, 1.48) [480]	**1.27** **(1.03, 1.56)** **[478]**	1.14 (0.94, 1.38) [554]	**1.20** **(1.00, 1.45)** **[552]**
Victimization ever (sum; continuous)	**1.05** **(1.01, 1.10)** **[515]**	**1.09** **(1.05, 1.14)** **[514]**	1.02 (0.96, 1.08) [335]	**1.10** **(1.04, 1.17)** **[334]**	1.03 (0.97, 1.08) [272]	1.04 (0.99, 1.09) [271]
Physically hurt you?	**1.32** **(1.12, 1.55)** **[524]**	**1.44** **(1.24, 1.6753)** **[523]**	0.97 (0.78, 1.20) [338]	**1.32** **(1.07, 1.64)** **[337]**	1.14 (0.94, 1.39) [278]	**1.21** **(1.01, 1.45)** **[277]**
Insulted or talked down to you?	1.08 (0.90, 1.29) [521]	**1.22** **(1.03, 1.44)** **[520]**	1.02 (0.80, 1.29) [338]	**1.23** **(1.00, 1.51)** **[337]**	1.06 (0.86, 1.32) [278]	1.08 (0.88, 1.32) [277]
Threatened you with harm?	**1.21** **(1.02, 1.43)** **[523]**	**1.26** **(1.08, 1.47)** **[522]**	1.17 (0.95, 1.44) [338]	**1.37** **(1.13, 1.66)** **[337]**	1.00 (0.81, 1.23) [280]	1.02 (0.84, 1.25) [279]
Screamed or cursed at you?	1.20 (0.99, 1.45) [523]	**1.26** **(1.05, 1.50)** **[522]**	1.17 (0.90, 1.52) [338]	**1.40** **(1.11, 1.77)** **[337]**	1.13 (0.90, 1.42) [279]	1.12 (0.91, 1.38) [278]
Restricted your actions?	1.16 (0.98, 1.38) [522]	**1.26** **(1.08, 1.47)** **[521]**	1.14 (0.92, 1.42) [337]	**1.26** **(1.03, 1.54)** **[336]**	1.15 (0.95, 1.41) [279]	1.16 (0.97, 1.40) [278]
Had non-consensual sex? (i.e., had sex when they did not agree to and/or want to, or were forced to)	0.93 (0.70, 1.24) [520]	1.19 (0.92, 1.54) [519]	0.68 (0.42, 1.11) [336]	0.85 (0.51, 1.43) [335]	0.92 (0.66, 1.27) [278]	1.02 (0.74, 1.40) [277]

*RR* relative risk. Bold text indicates *p* < 0.05. *Adjusted for age, gender, and education

The victimization scale had a large amount of missingness as participants were first asked if they were willing to share their experiences and hundreds of participants declined. Victimization (both the total and all components except for non-consensual sex) is associated with history of incarceration in Toronto and London, but this result was not significant in Thunder Bay.

## Discussion

This is among the first Indigenous-driven studies to examine colonialism and ever been incarcerated among First Nations, Inuit, and Métis people in London, Thunder Bay, and Toronto, Ontario, Canada. It is important to study Indigenous people living in urban settings because there is a dearth of reliable data on this group. Having reliable and valid data for each city will assist with receiving appropriate funding levels and having appropriate policy and legislation. This study is crucial because it provides a current picture of Indigenous people living in urban settings in Ontario.

Overall, we found that the proportion of *ever being* incarcerated was extremely high for Indigenous people living in these three urban settings. These proportions were higher than government statistics because those statistics only examine how many Indigenous people are incarcerated in a given year, without regard to its cumulative effect. Examining ever being incarcerated allowed us to highlight how much incarceration affects Indigenous people in their lifetimes.

The proportions of CPA involvement as children were higher for Indigenous people in Toronto who had a history of incarceration, while residential school attendance and Sixties Scoop proportions were higher in the incarceration history group in Thunder Bay. In Thunder Bay, nearly 20% of all participants reported residential school attendance (twice that of Toronto and nearly seven times that of the London sample), highlighting diversity in colonial experiences and associated violence. Family separation in Thunder Bay was more frequently due to residential school attendance and Sixties Scoop rather than the result of CPA involvement. Therefore, policy and funding need to be allocated to fit the profile of each community. For example, in Thunder Bay, more services and funding may need to support families and communities dealing with residential school impacts and family disruption.

There was variation in employment and education experiences across the three communities as well. Individuals with a history of incarceration in Thunder Bay and Toronto had higher levels of education than those who had no history of incarceration. Further, those in Thunder Bay who had a history of incarceration generally reported current employment. This may reflect the diversity in incarceration experiences, including access to education and employment programs. Unfortunately, the data do not include whether Indigenous people had access to education and employment programs when they were incarcerated.

In Toronto and Thunder Bay, people with a history of incarceration had higher proportions of victimization compared to the group with no history of incarceration. This is consistent with other findings that incarcerated Indigenous women had high proportions of victimization (De Ravello et al., 2008). The victimization scale had a very large amount of missingness as participants were first asked if they were willing to share their experiences and hundreds of participants declined. OHC Thunder Bay had the largest proportion of missingness in this section. This may be a case of non-ignorable missingness relative to this predictor, where a large proportion of participants who had these experiences did not feel comfortable answering these questions in Thunder Bay.

Systematic racism and discrimination in Canada and the justice system play a role in the high prevalence of incarceration among Indigenous peoples. For example, Indigenous youth received longer incarceration sentences regardless of factors such as their criminal history and offense severity compared to non-Indigenous youth (Latimer & Foss, 2004). This is consistent with experiences of Indigenous peoples in other colonial contexts. For example, approximately 80% of non-Indigenous youth in the justice system in Australia were diverted from court, in contrast to 45% of Indigenous youth, meaning 55% of Indigenous youth were going to court (Harker, 2010 cited in Blagg, 2012).

We did not identify residential school attendance as an increased risk of incarceration. This may have been due to confounding and a lack of power. It is possible that residential school attendance by parents or extended family members increased the risk for child protection experience, which in turn adds risk for incarceration, and thus could be a cascade effect through the active mechanisms of colonialism. There is limited research on this association, but more research, such as interviewing Indigenous adults who have had these experiences, is needed to better understand these associations. Again, we did not pool the data for these regressions as each study site wanted statistical analyses done for their community.

### Study limitations

This study has a number of strengths and limitations, with a strength being that it is among the first Indigenous-driven studies to examine colonialism and incarceration across three urban centres in Ontario. However, there were no available data around the types of crimes committed. Having more information on the types of offenses (e.g., drug offenses, property damage, violent/not violent), severity of offenses, and number of offenses that occurred may provide greater insights. Moreover, there are no data on Indigenous people who have not been incarcerated but may have had other experiences with the justice system, such as probation. Another limitation is the cross-sectional nature of the study which does not allow for full causal pathways. Incarceration and foster care were experienced in the past, but other variables, such as racism, victimization, and homelessness, may have occurred before and/or after incarceration.

## Conclusion

The high prevalence of incarceration among First Nations, Inuit, and Métis people in London, Thunder Bay, and Toronto highlights the urgent need for major reform, outlining racialized inequities through the Canadian justice system. Such racialized inequities are challenging—perpetuating and manufacturing health and well-being harms, with Indigenous peoples urgently requiring further supports in the form of funding and programming to help decolonize the legal system. This requires an in-depth understanding of colonial impacts, including but not limited to experiences of racism, victimization, and family disruptions (past and present) to inform policy and funding for Indigenous peoples in urban settings to reduce overrepresentation of Indigenous peoples in the Canadian justice system. Further, future research in this area should include examining Indigenous methods of justice such as restorative justice alongside Indigenous community partners.

## Contributions to knowledge

What does this study add to existing knowledge?

- This study is the first Indigenous-led study to examine and report that victimization, foster care, and racism are associated with ever being incarcerated.
- This study also highlights that systems issues (e.g., racism, foster care) need to be addressed to reduce the overrepresentation of Indigenous people in the legal system.

What are the key implications for public health interventions, practice, or policy?

- First, the numbers of Indigenous children going into foster care need to be reduced. Second, the effects of racism and victimization need to be highlighted and levels of racism and victimization need to fall as well.
- Public health interventions need to focus on supporting Indigenous people by *working with them*.
- Policy needs to address all three of the above issues in order to reduce suffering, but also to reduce levels of incarceration for Indigenous people.

## Data Availability

Data are owned by the three Indigenous community partners.
